# Video-assisted subtotal parietal pleurectomy: an effective procedure for recurrent refractory pneumothorax

**DOI:** 10.1186/s12893-022-01653-5

**Published:** 2022-05-26

**Authors:** Haomin Cai, Rui Mao, Yiming Zhou

**Affiliations:** Department of Thoracic Surgery, Shanghai Pulmonary Hospital, Tongji University School of Medicine, 507 Rd Zhengmin, Yangpu District, Shanghai, 200433 China

**Keywords:** VATS, Pleurectomy, Pneumothorax

## Abstract

**Background:**

Refractory pneumothorax combined with diffuse emphysematous changes is an intractable problem requiring surgical treatment. Traditional bullectomy may result in long-term air leakage and has a risk of early recurrence. Pleurectomy is an effective pleurodesis procedure, which appears to be more suitable for these cases. We conducted this study to present our experience with this procedure.

**Methods:**

We collected the clinical data of 14 patients who underwent subtotal pleurectomy via video-assisted thoracic surgery (VATS) in our institution from November 2016 to October 2021. All patients had undergone complete preoperative examinations and met the indications for pleurectomy. Regular follow-up was conducted after surgery.

**Results:**

The study population was composed of 11 males and 3 females, with an average age of 52.4 ± 19.0 years. Subtotal pleurectomy via VATS was successfully performed in all patients, with no conversion to open surgery. The average operation time was 82.5 ± 23.4 min (range 45–120 min), intraoperative blood loss was 92.9 ± 37.1 mL (range 50–200 mL), postoperative hospital stay was 5.0 ± 4.8 days (range 2–19 days), and chest tube duration time was 22.1 ± 13.0 days (range 5–49 days). No major complication occurred except for one case in which reoperation was performed due to massive postoperative hemorrhage. The mean follow-up time was 24.8 ± 17.0 months (range 6–60 months); no recurrence was noted.

**Conclusions:**

Subtotal pleurectomy via VATS is a satisfactorily effective procedure for preventing pneumothorax recurrence.

**Supplementary information:**

The online version contains supplementary material available at 10.1186/s12893-022-01653-5.

## Introduction

Refractory pneumothorax has always been an intractable problem requiring surgical treatment. Unlike pneumothorax caused by an isolated pulmonary bulla, some types of pneumothoraces have associated comorbidities that make treatment very difficult [1]. The most common one is emphysema with multiple and diffuse pulmonary bullae. It is characterized by recurrent spontaneous pneumothorax that is difficult to manage with conservative treatment, such as prolonged drainage or even chemical pleurodesis. Although the British Thoracic Society [2] and the German S3 [3] guidelines recommend elective surgical pleurodesis for a non-resolving pneumothorax to prevent recurrence, the detailed pleurodesis procedure has not been specified. Bullectomy, whether via the traditional open technique or by video-assisted thoracic surgery (VATS), is not suitable for such patients due to incomplete removal of all bullae and a risk of long-term persistent air leakage. We describe our process of subtotal pleurectomy via VATS without bullectomy performed for 14 such patients, which has proven effective with no recurrence during follow-up.

## Materials and methods

### Patients

This retrospective analysis was approved by the institutional review board of Shanghai Pulmonary Hospital, and there was no registration of this study. Patients who underwent subtotal pleurectomy via VATS by one surgeon (Yiming Zhou) in the Department of Thoracic Surgery from November 2016 to October 2021 were enrolled. The criteria for inclusion in the study were: (1) patients aged 16–75; (2) diagnosis of persistent pneumothorax (air leak lasted longer than 7 days after non-surgical treatment); (3) Chest computed tomography (CT) presented as diffuse emphysematous change or absence of exact lesion that are highly suspected of causing air leakage. The patients enrolled were analyzed according to the intention-to-treat approach, and a total of 17 cases met inclusion criteria. Among them, 3 were excluded due to loss to follow-up (n = 2) or undergoing bullectomy during surgery (n = 1). Ultimately, 14 patients were included in the analysis. Data were collected from the patients’ medical records.

### Preoperative preparation

All patients were suffered from persistent air leakage and underwent conservative treatment including closed thoracic drainage, respiratory physiotherapy, and suction of drainage collection chamber. Every patient had to undergo a routine laboratory examination, including blood and coagulation tests, electrogram, and cardiac ultrasound to assess the tolerance to general anesthesia. A pulmonary function test could not be performed due to the pneumothorax. High-resolution computed tomography was performed to assess if the bullae were diffusely distributed and if the patients were good candidates for subtotal pleurectomy via VATS.

### Surgical technique

**Fig. 1:**
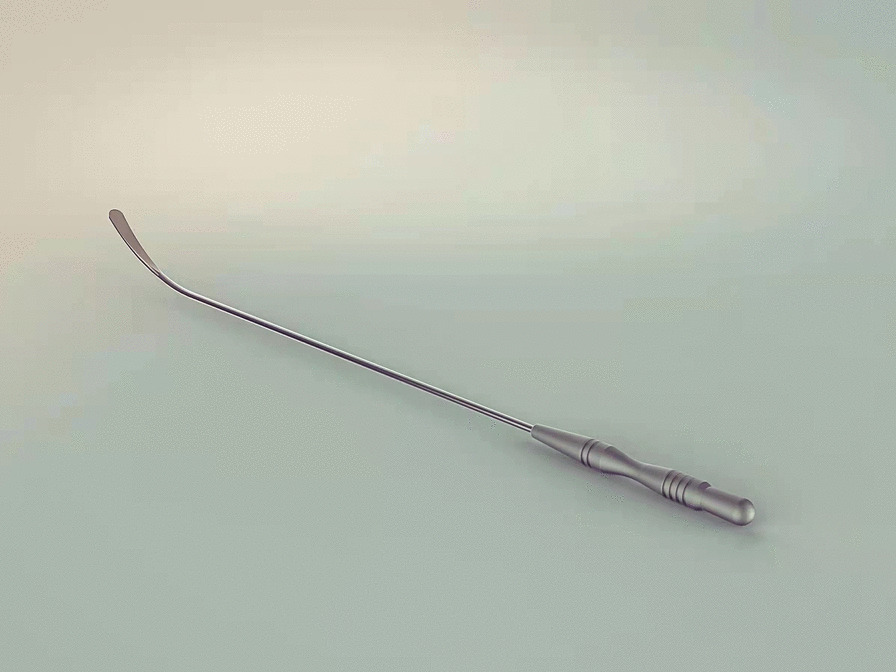
The self-designed endoscopic stripper for dissecting the parietal pleura

**Fig. 2:**
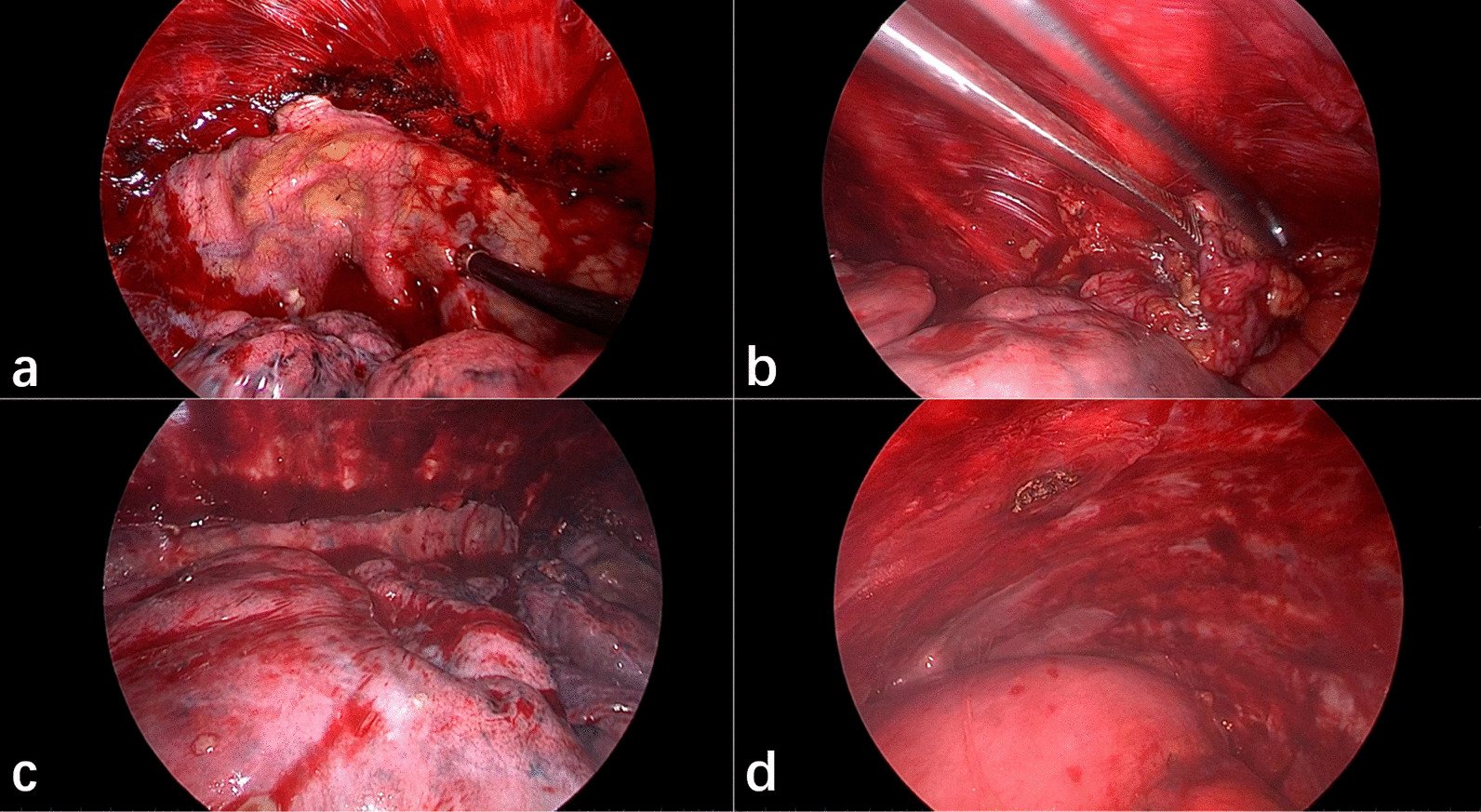
The resection range of subtotal pleurectomy via VATS. **a** Up to the first rib; **b** to the side of the sternum anteriorly; **c** to approximately 1 cm from the sympathetic chain posteriorly; **d** down to the level of the diaphragm. *VATS* video-assisted thoracic surgery

All surgeries were carried out under general anesthesia and double-lumen endotracheal intubation, and in the lateral decubitus position. Single-lung ventilation was applied during the intervention. The thoracic cavity was entered via an incision approximately 4 cm long in the 5th intercostal space at the anterior axillary line. A 1-cm incision was occasionally added for the camera trocar in the 7th intercostal space at the midaxillary line. The chest cavity was inspected, and loose adhesions were easily separated. On the other hand, dense adhesions were untouched to avoid massive hemorrhage and lung injury. A pair of scissors was used for separating the parietal pleura and entering the extrapleural space around the operative incision. We then used a self-designed stripper (Fig. 1), which was described in our previous study about uniportal VATS decortication for tuberculous empyema [4], to further dissect bluntly the surrounding pleura. The parietal pleural dissection edge extended to the first rib, down to the level of the diaphragm, anteriorly to the side of the sternum, and posteriorly to approximately 1 cm from the sympathetic chain (Fig. 2). The pleura on the diaphragm may be left intact. When the parietal pleura in the aforementioned areas has been resected, hemostasis was performed and electrocautery was used to control bleeding from the small arteries on the chest wall, which were mostly found in the areas where there were adhesions. After the operation, a 28-Fr chest tube was inserted to drain the upper part of the chest and connected to a water seal system (Additional file 1: Video S1).

### Postoperative therapy

Postoperative pain was generally managed with an analgesia pump (sufentanil citrate) during the first 24 h, followed by regular intake of ibuprofen. Patients who met the criteria (Caprini score ≥ 3 [5] and amount of drainage < 500 mL during the first 24 h) received preventive anticoagulation therapy with low-molecular-weight heparin. The chest radiograph was reviewed on the 1st day after the operation. If lung recruitment was not sufficient, the digital drainage system with vacuum suction was applied. Patients with restoration of the preoperative status, satisfactory lung recruitment, and no occurrence of significant complications could be discharged. Patients with a small amount of persistent air leakage were discharged, with the chest tube in place until the leakage disappeared and weak fluctuation was observed in the water column of the drainage system.

### Follow-up

**Fig. 3:**
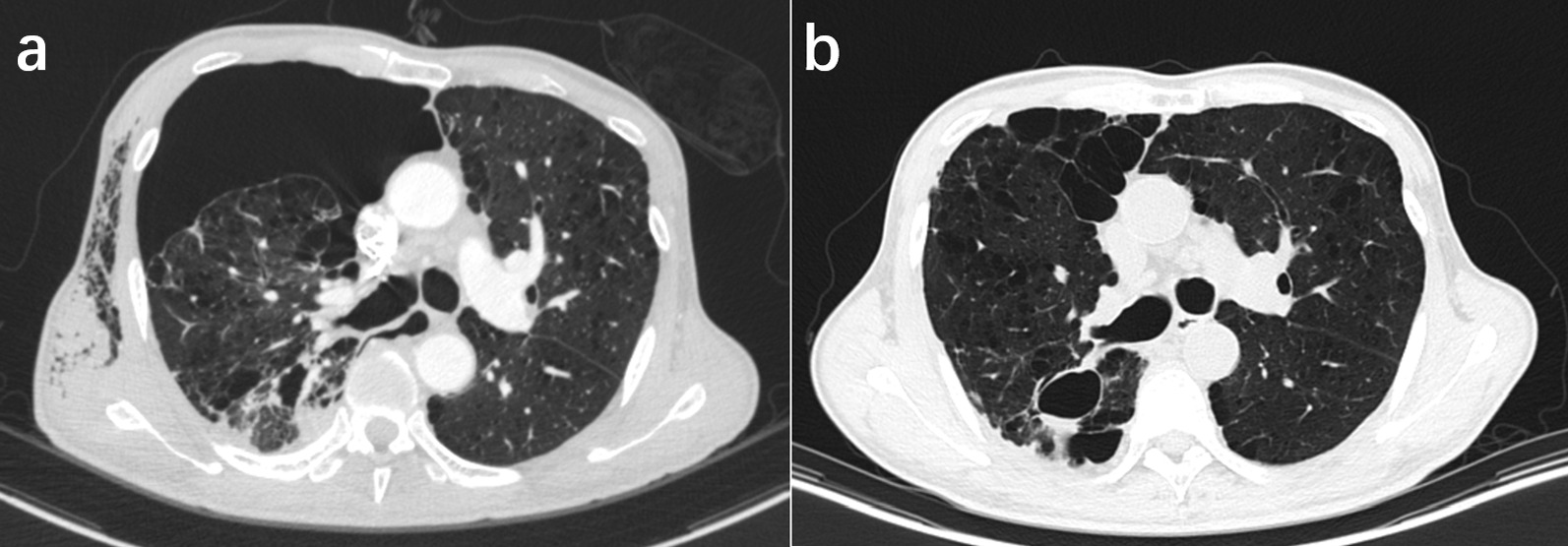
Comparison of preoperative and postoperative computed tomography (CT) scans. **a** Preoperative CT scan shows right lung collapse with diffuse emphysematous changes and multiple blebs; **b** Follow-up CT scan after 6 months shows total lung recruitment and no recurrence of pneumothorax

Outpatient or telephone follow-up was carried out at 3 weeks and then every 6 months after surgery. The surveillance CT scans every 6 months were used to evaluate the surgical outcome (Fig. 3).

## Results

This study included 11 male and 3 female patients, with a mean age of 52.4 ± 19.0 years (range 19–74) and a mean body mass index (BMI) of 21.4 ± 2.7 kg/m^2^ (range 15.6–26.6 kg/m^2^). One female case was clinically diagnosed with catamenial pneumothorax, while for the rest of the cases, emphysematous changes were seen in the ipsilateral lung on preoperative CT scan. The sign of pleurodesis, such as pleural thicking or calcification was not observed in contralateral chest cavity. All cases did not resolve with conservative treatment over 10 days, and 13 of them suffered from recurrent pneumothorax. Three of the cases had a history of having undergone bullectomy in other hospitals. All patients successfully underwent subtotal pleurectomy via VATS. No case was converted to open surgery. Nearly all emphysematous change was secondary to chronic obstructive pulmonary disease (COPD), except for one case who was highly suspected lymphangiomyomatosis (LAM) preoperatively, underwent a small wedge resection for pathological examination. The average operation time was 82.5 ± 23.4 min (range 45–120 min). No patient received blood transfusion during the operation and the average intraoperative blood loss was 92.9 ± 37.1 mL (range 50–200 mL). The average drainage volume was 510.7 ± 269.4 mL (range 100–1200 mL) on the first day after the operation. Massive postoperative hemorrhage occurred in one patient. The drainage volume reached 1200 mL 4 h after the operation; thus, reoperation was performed through the original incisions. The source of the arterial bleeding was localized on the chest wall at which there had been dense adhesions; hemostasis was then completed by electrocoagulation. The average postoperative hospital stay was 5.0 ± 4.8 days (range 2–19 days). Thirteen patients had prolonged air leakage (> 5 days), with an incidence rate of 92.9%. No other major postoperative complications were registered. Time to chest tube removal was 22.1 ± 13.0 days (range 5–49 days) on average.

The postoperative follow-up time was 24.8 ± 17.0 months (range 6–60 months). There was no recurrence of pneumothorax in any of the patients, and lung recruitment was satisfactory as per imaging review (Fig. 3).

## Discussion

When performing surgical treatment for secondary pneumothorax combined with diffuse emphysema and COPD, it is quite common that the sources of the air leak cannot be identified during the surgery, making bullectomy impossible. Thus, the treatment approach needs to shift to the obliteration of the pleural space in order to avoid recurrence of pneumothorax and achieve control. The obliteration of the pleural space mainly depends on the methods of pleurodesis. Pleurodesis may be chemical or mechanical. The drugs used for chemical pleurodesis include povidone–iodine, tetracycline, talc powder, and talc slurry, to name a few. Among these, talc powder is the easiest to evenly distribute throughout the chest cavity, especially during VATS, in which there is good intraoperative visibility and fixation, resulting in satisfactory pleurodesis. However, because of the risk of mixing asbestos in the production of talc, which may be potentially carcinogenic [6], its clinical application has been gradually reduced in recent years. Meanwhile, mechanical pleurodesis includes pleural abrasion and pleurectomy. Several studies have found that compared with pleural abrasion, even though the surgical procedure of pleurectomy is more complicated, pleurectomy has still advantage in controlling pneumothorax recurrence [7, 8], and it might have similar outcomes as talc pleurodesis [9].

Agata et al. found that for primary spontaneous pneumothorax with Vanderschueren classification ≥ stage III [10], pleurectomy without wedge resection has the advantage of lower recurrence compared to pleurectomy with wedge resection [11]. We obtained the same conclusion. For lungs with emphysema-like changes, it is difficult to identify intraoperatively the exact lesions causing the air leak; in most cases, we can usually only identify and resect the most suspicious one, or sometimes we simply perform a blind apical wedge resection. Unfortunately in these patients, the most severe emphysema, but not necessarily the source of the air leak, is often located at the apical area. Studies have found that bullectomy is the only independent risk factor for long-term postoperative air leakage [12]. If the stapler margin is too close to the root of the bulla during resection, the bullae easily regenerates, which could result in recurrence [13]. Therefore, we believe that unless the indications are clear and the resection margins all fall on normal lung tissue, bullectomy is not necessary.

We use the term “subtotal parietal pleurectomy” for our procedure because not all parietal pleura is resected. The pleura in the apex (above the first rib) is retained to avoid damage to important neurovascular tissue structures. It has been reported that only apical pleural abrasions can cause pseudoaneurysms [14]. The pleura on the diaphragm is likewise not removed because of the possibility of bleeding and its minimal usefulness in closing the pleural cavity. With regard to the posterior parietal pleura, the resection range was always kept at least 1 cm from the sympathetic nerve chain. We believe that excessive removal of the posterior pleura close to the vertebral column has little influence on generating lung adhesions. Moreover, pleurectomy may damage the sympathetic nerve chain on the T2–T4 level, which may cause palmar anhydrosis and compensatory contralateral hyperhidrosis, causing great distress to the patient [15].

Autologous blood was previously considered a safe, effective, and economical pleurodesis agent [16]. For our study, there was inevitably some blood oozing from the chest wall, which was not electrocoagulated unless massive bleeding or spurting was identified. We did not irrigate the surgical field to allow the residual blood to further promote pleurodesis. With the recruited lung compressing the chest wall after surgery, minor bleeding often stops spontaneously. One patient in our study underwent reoperation because of massive hemorrhage. This patient had a history of VATS pleural abrasion, and there were already dense adhesions during the operation. An aberrant artery behind the adhesions on the posterior chest wall led to massive bleeding after surgery. Therefore, we recommend that electrocoagulation should be much more carefully employed on areas with vessel-rich adhesions.

Negative pressure suction was applied after the operation, which promotes lung recruitment and allows the pleural cavity to close as soon as possible. In our series, the average time to chest tube removal is was 22.1 ± 13.0 days after surgery, which was obviously longer than most of other lung surgeries such as lobectomy. The reason is that although most cases no longer had air leakage within two weeks, the chest tube was kept until weak fluctuation was observed in the water-sealed drainage system, which meant almost total closure of the pleural cavity and potential benefit of reducing the risk of recurrence. Certainly, this is only our own experience which need more clinical evidence for further confirmation.

Our procedure of subtotal pleurectomy causes dense adhesions in the entire chest cavity. However, if lung surgery is necessary in the future, it will greatly increase the difficulty of separating the adhesions. In our study, one case was clinically suspected to have LAM. After adequate preoperative discussions, the patient refused to undergo lung transplantation because of financial reasons; hence, pleurectomy was performed instead.

There were obviously several limitations to our study. First, it was a single-institution retrospective study and the number of patients was small. Although our surgical group performed 101 pneumothorax cases during the study period, most of them were not presented as diffuse bullae and underwent conventional bullectomy and pleural abrasion, finally only 14 cases were enrolled. A well-designed, prospective randomized controlled trial is needed to further validate the outcome of this procedure. Second, our study had a relatively short follow-up time. Past studies have found that recurrence long after pleurectomy is possible, with one of the possible mechanisms being the formation of a neopleura [17]. Although no recurrence was noted, the long-term effects of subtotal pleurectomy remain to be determined for our study population. In conclusion, our study indicated that subtotal pleurectomy via VATS is safe, with a low incidence of complications, and is satisfactorily effective in preventing recurrence of refractory pneumothorax.

## Supplementary Information

**Additional file 1: Video S1.** Video-assisted right subtotal parietal pleurectomy.

## Data Availability

The data used or analysed during the current study are available and can be obtained from the corresponding author upon reasonable request.

## Abbreviations

VATS: Video-assisted thoracic surgery
CT: Computed tomography
COPD: Chronic obstructive pulmonary disease
LAM: Lymphangiomyomatosis
